# Getting Lost in History: Mabel Purefoy FitzGerald and the Origins of Hydrochloric Acid in the Gastric Mucosa

**DOI:** 10.1093/function/zqab054

**Published:** 2021-10-20

**Authors:** Martha C Tissot van Patot

**Affiliations:** Fort Collins, CO, USA

## Introduction

A recent editorial by Ole H. Petersen, *When a discovery is a rediscovery: Do we know the history of our own subject?*, stimulated me to bring to light the work of Mabel Purefoy FitzGerald (1872–1973, Figure 1).^1^ In the process of writing FitzGerald's biography, I rediscovered that she was the first to prove the origin of hydrochloric acid (HCl) in the parietal cells of the gastric mucosa in 1910.^2,3^ Curious as to why her work is not cited for this achievement, I followed the trail of scientific literature that led to the dismissal of her contribution (Figure 2). FitzGerald's name may be familiar, as Sir Peter Ratcliffe recently acknowledged her contribution to the field of hypoxia research in his lecture accepting the 2019 Nobel Prize for Physiology and Medicine along William Kaelin and Gregg Semenza.

**Figure 1.**
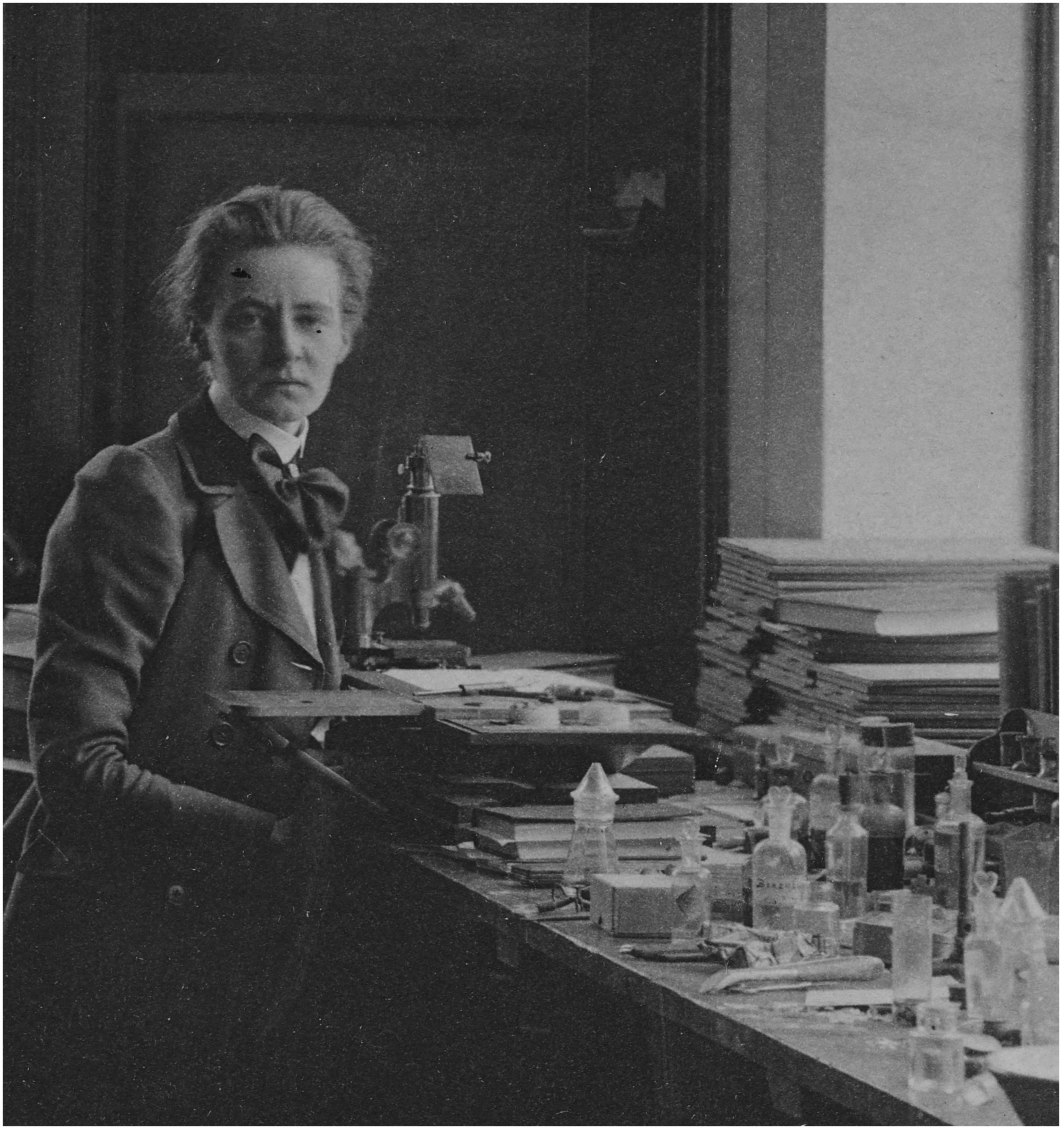
Mabel Purefoy FitzGerald in the laboratory of Carl J. Salomonsen, Copenhagen, 1902. Courtesy of Geoffrey Purefoy and the Bodleian Library Special Collections, Oxford, UK.

**Figure 2.**
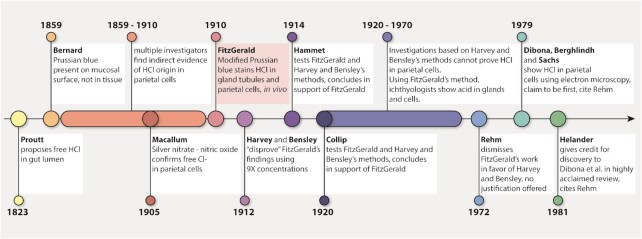
Timeline of events related to FitzGerald's work and subsequent dismissal. Designed by Coni Hoerndli, chsciencedesign.com.

* * * * *

“She has worked for the pure love of science and in the most disinterested spirit. I feel sure… that she not only will do her best, but that she will do really good work.”^4^

Archibald B. Macallum, first Professor of Biochemistry at the University of Toronto, was reading these words in 1908 in reference to Mabel Purefoy FitzGerald, who was applying to work in his laboratory. They were penned by the exacting and demanding microchemical histologist Gustav Mann. FitzGerald trained with Mann in Oxford from 1897 to 1902, while he labored to complete his *Methods and Theory of Physiological Histology*, lauded by Nature as the first “coherent and harmonious” text of “physical chemistry colloids, histology, and the chemistry of dye-stuffs.”^5^

Macallum was looking for someone with an expertise in microchemical histology to assist him in proving that HCl was formed in the parietal cells of the gastric mucosa. A total of 3 yr earlier, he confirmed the findings of Mary Greenwood of Girton College, Cambridge, that chloride was present in parietal cells.^6^ She used silver nitrate to precipitate the chloride, but it then became apparent that phosphate and carbonate could also cause precipitation. Macallum modified Greenwood's method with nitric acid so that the reaction would only occur in the presence of chloride and verified her conclusion.^7^ He was now eager to prove that HCl was present in parietal cells.

FitzGerald recently completed the first 9 mo of a traveling fellowship awarded to her by the newly established Rockefeller Institute in New York City. She had not had a positive experience and was eager to finish her fellowship in a new location. FitzGerald's friend and colleague Maud Menten (soon to develop the Michaelis–Menten equation^8^) was returning to Toronto to earn her medical degree and suggested that FitzGerald apply to work in the lab of her former mentor, Professor Macallum.

**Figure 3.**
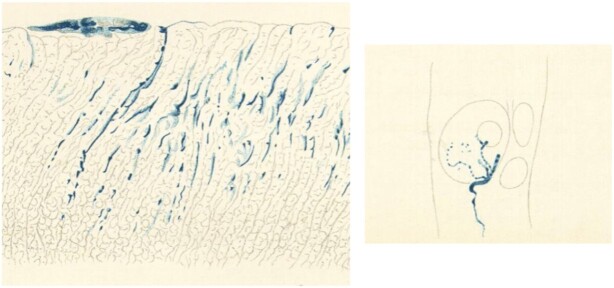
Prussian blue staining the central lumina of gland tubules, in crypts and lining of the gastric mucosa (left, X96), and parietal cell and canaliculi (right, x1100) in a rabbit. Adapted from FitzGerald MP. *Proc R Soc Lond B Biol Sci* 1910;83 (561):56–93.

Macallum responded with alacrity, requesting that FitzGerald please come as soon as possible because 3 mo was not much time to accomplish anything significant. FitzGerald arrived in October and by midDecember proved the presence of HCl in parietal cells (Figure 3). An accomplishment that had eluded investigators for 50 yr.

Thrilled with her success, Macallum took FitzGerald to her first meeting of the American Physiological Society in Baltimore where he presented her findings. Only members could present at the meetings. This was nothing new to FitzGerald, she was not permitted to present any of her work throughout her career.

He insisted FitzGerald be the sole author on the 2 resulting publications. The first was a brief report published in May 1910, quickly followed by a lengthy manuscript in which she not only presented her data but provided a detailed history of the work of preceding investigators that led to her success.^2,3^

The manuscript begins with William Proutt's hypothesis in 1823 that free HCl exists in the gut lumen and jumps to an 1859 study by Claud Bernard, the father of experimental physiology, who found evidence of acid not in the cells but on the surface of the gastric mucosa. FitzGerald then details extensive empirical evidence published by Brücke,^9^ Heidenhain and Rollett,^10,11^ Frankel^12^, and others over the ensuing 50 yr refuting and supporting Bernard's findings.

She then provides evidence that Bernard's initial Prussian blue method was flawed because the chemicals used (lactate of iron and potassium ferrocyanide) are capable of forming a light blue color over time without the presence of acid. With her extensive knowledge of microchemical methods, FitzGerald solves this dilemma by making ammonium ferric citrate to replace the lactate of iron. This forms a more stable chemical combination that will not change color unless a free acid is added. The resulting histology revealed Prussian blue staining in parietal cells and canaliculi of the gastric mucosa. This is the first definitive proof that HCl originates in parietal cells, yet FitzGerald is not credited with the discovery.

In 1912, Harvey and Bensley claimed to have repeated FitzGerald's experiments but with differing results and concluded that her work was flawed and free acid was not present in parietal cells.^13^ Hammet, after reviewing the findings of each manuscript and testing the methods, reported that he obtained “the recorded results and a stable confirmation of Miss FitzGerald's experiments and conclusions.”^14^

In 1920, Collip further investigated the discrepancy between the 2 publications. FitzGerald extensively tested and defended her use of 2.25% ammonium ferric citrate and 1.5% sodium ferrocyanide, whereas Harvey and Bensley used 25% and 10% concentrations without justification and reported extensive Prussian blue staining throughout the body. Collip found that these “exceedingly” high concentrations formed “copious precipitates of Prussian blue” in the absence of acid. He further concluded that the blue stain present in other organs was likely a result of cells releasing lactate in response to the toxic effects of the high salt concentrations.^15^

Despite Hammet's and Collip's conclusions, investigators continued to employ Harvey and Bensley's methods with poor results.^14,16–20^ In an odd twist of history, ichthyologists preferred FitzGerald's methods and reported great success.^21–24^

In 1972, Rehm reviewed the topic and dismissed FitzGerald's work in favor of Harvey and Bensley without justification.^2,13,25^ In 1979, Dibona, Berghlindh, and Sachs provided evidence for the presence of HCl in parietal cells using electron microscopy. They cite Rehm and repeat his assertion that while “it is generally assumed that the parietal cell is responsible for acid secretion by the stomach, exact localization of the process has not been previously demonstrated in intact tissue.” ^25,26^

In 1981, Helander wrote a comprehensive review titled *Cells of the Gastric Mucosa*, giving credit to Dibona, Berghlindh and Sachs for providing the first proof that HCl is produced in parietal cells, also citing Rehm's paper as evidence of no previous success in this area.^25–27^ His review has been cited 225 times, thus solidifying the descent of FitzGerald's work into obscurity.

There is a term popularized by Sir Isaac Newton to the effect that we “stand on the shoulders of giants” in our research endeavors. Written in the “most disinterested spirit,” FitzGerald's recognition of extensive work by numerous investigators leading to her success, reveals the inherent fallacy of this popular phrase. Our success cannot be attributed to a few giants, rather it builds on the steady careful work of many, the sum of whose innumerable contributions to our collective knowledge have inexorably pushed us forward.

*This work was supported in part by the Paton Prize Bursary awarded by The Physiological Society, London, UK.
